# O21 A rare case of anti-TNF induced panniculitis

**DOI:** 10.1093/rap/rkaa054.009

**Published:** 2020-11-03

**Authors:** Alaeldin Mohamednour, Sanna Khan, Kehinde Sunmboye, Alison Kinder

**Affiliations:** Leicester Royal Infirmary, Leicester, United Kingdom

## Abstract

**Case report - Introduction:**

Panniculitides comprise a heterogeneous group of inflammatory diseases involving the subcutaneous fat. They remain the most challenging areas for clinicians. Skin biopsy is commonly needed to confirm diagnosis. Because there are many underlying aetiologies for panniculitis, detailed history and thorough investigations are needed. We present a case of A 20-year male who was admitted with painful lumps treated initially as cellulitis/abscess but turned to be neutrophilic panniculitis on skin biopsy. Extensive workup failed to reveal underlying aetiology. Eventually Imradli (AntiTNF) was thought to be the culprit and therefore was kept on hold with no recurrence of panniculitis.

**Case report - Case description:**

A 20-year-old, Asian Malawian. Moved to the UK at the age of 6. He was diagnosed with Ankylosing spondylitis in November 2016. Initially received Naproxen followed by (Humira) with good clinical response. He was switched to biosimilar Imradli in Nov 2019. He was admitted with 2–3 weeks history of progressive right hip and buttock pain, 1 week of very tender erythematous swelling of the right buttock but without fever or weight loss. He reported mild weakness of lower limbs. Physical examination revealed 5x 8 cm swelling on Right buttock, Rest of examination was unremarkable. He was reviewed by neurology team who arranged MRI spine and brain, EMG and lumbar puncture which all came back as unremarkable excluding the possibilities of myelitis and myositis. Initially thought to be abscess/cellulitis but absence of fever/inflammatory response, abnormal CT finding and no response to antibiotics made it less likely. While the Right buttock erythema/swelling started to resolve, he developed two new migratory erythematous lesions appearing around the left buttock and lower lumbar spine. Working diagnosis of panniculitis was made which was confirmed on biopsy. Due to lack of response to NSAIDs, colchicine or oral steroids, a 3rd biopsy of the freshest lesion was performed to exclude deep-seated infection.

Investigations –

FBC, U&ES, LFT, CRP, CK, ACE - all were unremarkable

ASO titre <200, serology for Borrelia and TPHA negative.

Viral, parasitic, and Autoimmune screen were unremarkable.

CXR clear, MRI/CT: extensive subcutaneous inflammatory changes in the right buttock with sacral oedema.

PET-CT – showed resolving inflammatory changes in the right flank, FDG intake in C6 and SI joints presumed secondary to ankylosing spondylitis and sacroiliitis.

The underlying cause of panniculitis remains uncertain. Anti TNF was kept on hold and the patient was followed up with no evidence of recurrence of panniculitis

**Case report - Discussion:**

Panniculitis (inflammation of subcutaneous fat) is a relatively uncommon condition. It has various aetiologies including infection, trauma, inflammation, and malignancy. Skin biopsy can give valuable information including microbiological studies if infectious panniculitis was suspected. However, clinical correlation and careful consideration of the differential diagnosis is needed in many cases.

The diagnosis can be quite challenging as in this case where all investigations and skin biopsy could not point towards the underlying aetiology. Although anti-TNF inhibitors are commonly used in treating a wide range of autoimmune conditions. But their use can lead to the development of secondary autoimmune diseases, such as cutaneous vasculitis, lupus-like syndrome, and interstitial lung disease, paradoxically induced by anti-TNF-α agents. Llamas-Velasco and Requena, reported the first case of panniculitis induced by etanercept injection in a 62-year-old woman with severe psoriasis who developed an erythematous, slightly painful nodule on the skin of the anterior abdominal wall.

Adalimumab induced lupus panniculitis was reported in a Rhu-lupus patient. Although the lesions stopped progressing after cessation of adalimumab, they remained unchanged for two more years. The mechanism for adalimumab-induced CLE is uncertain.

Although there is not enough data about autoimmunity with biosimilars, we think secondary autoimmune conditions could similarly be induced by biosimilar as illustrated in this case. Anti-TNF induced cutaneous panniculitis is considered most likely although uncertain. If anti-TNF drug-induced, this should gradually resolve but can be slow (4–6 months). Corticosteroids have been added for an anti-inflammatory response, but there was little benefit which might point to a different pathogenetic mechanism.

NSAIDs has helped to keep his AS relatively stable during the COVID-19pandemic. During the last review, the patient expressed his wishes to go back on biologic. But the question remains whether he will a have a recurrence of panniculitis or not?

**Case report - Key learning points:**

1/Anti-TNF inhibitors sometimes cause secondary autoimmune conditions like cutaneous vasculitis, lupus-like syndrome, but there is not enough data regarding biosimilar induced autoimmunity.

2/This case illustrates the high importance of having a tissue diagnosis. (whenever there is an issue, the diagnosis would be in the tissue).

3/There is still uncertainty whether a recurrence of panniculitis might occur or not if the patient went again on biologics.

